# Particularities of suicide attempts among adolescents in the region of southern tunisia

**DOI:** 10.1192/j.eurpsy.2021.577

**Published:** 2021-08-13

**Authors:** A. Kerkeni, W. Abbes, M. Tfifha, W. Mahdhaoui, M. Abbes, I. Rejeb, L. Ghanmi

**Affiliations:** 1 The Department Of Psychiatry, Hospital of gabes, Gabes, Tunisia; 2 Psychiatry, regional hospital of Gabes, Gabes, Tunisia; 3 Emergency Department, Hospital of gabes, Gabes, Tunisia

**Keywords:** Suicide attempts, adolescents, Particularities

## Abstract

**Introduction:**

Adolescent suicide and suicide attempts (SA) present a complex and multifactorial problem that deserves special attention. Identifying particularities of suicidal behavior in this age group is essential in order to identify suffering adolescents.

**Objectives:**

To determine the characteristics of adolescent suicide attempts compared to those of adults.

**Methods:**

It was a retrospective study carried out on a clinical population who consult in the psychiatry department at the Gabes regional hospital during the period from January 1st, 2020 to September 30, 2020. Sociodemographic and clinical data of the patients as well as their family dynamics, their education, their personal and family history, characteristics of the SA and psychiatric symptoms preceding the act were assessed. We compared two subgroups: patients aged 10 to 19, and those aged 20 and over.

**Results:**

278 suicide patients were included. 101 of them were adolescents (10 -19 years), of which 89 (88.11%) were female. Mean age of suicidal adolescents was 16.5 years. They were mostly living with their families (92.07%). Intentional drug ingestion was more common in adolescents (81.1%) than in adults (40%). Adolescent suicide attempts were correlated with a conflictual family environment (p=0.04), exposure to mistreatment (p=0.001), the absence of underlying mental disorders (p<10-3), the presence of academic difficulties (p<10-3) and the presence of a precipitating factor such as family conflict (p<10-3) or school failure (p= 0.004).

**Conclusions:**

A good knowledge of the particularities of suicidal behavior in adolescents is preliminary to support an effective preventive measure targeting both family and school environment

